# The Association Between Opium Consumption, Diabetes, and Hypertension Among Male Participants in the Tabari Cohort Population: A Cross-sectional Study

**DOI:** 10.34172/jrhs.11343

**Published:** 2025-10-18

**Authors:** Keyvan Heydari, Motahareh Kheradmand, Somayyeh Ahmadnezhad, Mahmood Moosazadeh

**Affiliations:** ^1^Student Research Committee, School of Medicine, Mazandaran University of Medical Sciences, Sari, Iran; ^2^Health Sciences Research Center, Mazandaran University of Medical Sciences, Sari, Iran; ^3^Department of Emergency Medicine, Ramsar Campus, Mazandaran University of Medical Sciences, Ramsar, Iran; ^4^Gastrointestinal Cancer Research Center, Non-communicable Diseases Institute, Mazandaran University of Medical Sciences, Sari, Iran

**Keywords:** Blood glucose, Hypertension, Opium, Cohort studies

## Abstract

**Background:**

Opium consumption is a prevalent health concern in Iran, with conflicting evidence regarding its association with chronic conditions such as diabetes mellitus (DM) and hypertension (HTN).The present study aimed to investigate the association between opium consumption and the risk of diabetes mellitus (DM) and hypertension (HTN) among male participants aged 35–70 years in the Tabari Cohort Study (TCS).

**Study Design:**

A cross-sectional study.

**Methods:**

This study examined male participants from the TCS. Blood samples were collected after a 12-hour fasting period. HTN and DM were defined based on blood pressure (BP) measurements, history of diagnosis, and use of antihypertensive or glucose-lowering medications. The obtained data were analyzed using chi-squared and independent t-tests. A multivariate logistic regression analysis was used to adjust for potential confounders.

**Results:**

The study examined 4,149 male participants, with a mean fasting blood sugar (FBS) of 110.34±3 3.89 mg/dL, systolic BP of 115.70±13.60 mm Hg, and diastolic BP of 73.87±7.77 mm Hg. No significant difference was found in the frequency of HTN and DM among participants who consumed opium compared to those who did not consume it (*P*=0.588 and *P*=0.705, respectively). However, FBS levels were significantly higher among opium users (110.77±34.14 vs. 107.73±21.19, *P*=0.048). Multivariable regression analysis revealed no significant change in the risk of developing HTN (odds ratio [OR]: 1.06, 95% confidence interval [CI]: 0.82, 1.35, *P*=0.667) and DM (OR: 1.22, 95% CI: 1.57, *P*=0.116) among opium users.

**Conclusion:**

The findings of this study demonstrate no significant difference in the likelihood of developing DM and HTN between opium users and non-users.

## Background

 Illegal drug use remains a major global health concern, with opium being one of the most commonly abused substances, particularly in parts of Asia and the Middle East.^1^ Drug abuse refers to the use of illegal substances or the misuse of legal medications for nonmedical purposes.^2^ Despite a decline in its prevalence in recent years, opium consumption remains significant in Iran, affecting both younger and older age groups. Previous reports estimate opium abuse among 6% of men and 2% of women in the country.^3-6^

 Opium is derived from the poppy plant *Papaver somniferum* and contains over 20 different alkaloids, including morphine and codeine.^1^ It primarily affects the central nervous system but also influences the cardiovascular, respiratory, and endocrine systems.^7^ Studies have shown that opium can impact blood pressure (BP) regulation, possibly through effects on sodium and calcium channels and interference with enzymes, such as adenylate cyclase.^8,9^ These effects may contribute to both hypotensive and hypertensive states.

 Several studies have explored the relationship between opium use and chronic conditions, such as diabetes mellitus (DM), hypertension (HTN), and cardiovascular diseases. While some suggest that opium increases the risk of hyperglycemia and DM,^10-15^ others have reported no long-term effect or even temporary glucose-lowering outcomes.^16^ Misconceptions persist among some individuals with DM who believe opium can alleviate hyperglycemia and its associated symptoms, potentially contributing to its continued use.^12^ Additionally, opium use in patients with chronic diseases has economic consequences, including poor adherence to treatment and increased financial burden.^17-19^

 The prevalence of opium abuse, the burden of chronic conditions (e.g., DM and HTN), and the inconsistent findings in the literature underscore the need for further research. This study seeks to investigate the association between opium consumption and the risk of developing DM and HTN using data from a large population-based cohort in northern Iran.

 It has been hypothesized that opium consumption is associated with a higher risk of developing DM and HTN among male participants.

## Methods

###  Study design and population

 This was a cross-sectional study utilizing data collected during the enrollment phase of the Tabari Cohort Study (TCS). TCS is part of a national cohort study called the Prospective Epidemiological Research Studies in Iran (PERSIAN), which is performed in different parts of Iran. Overall, 180,000 Iranians, aged 35–70 years, from 18 geographically distinct areas in Iran were recruited in the first phase of the PERSIAN cohort study. TCS was conducted in the Mazandaran province of Iran. Mazandaran province is a district in northern Iran located in the foothills of the Alborz mountain range, including mountainous and plains. The capital city of Mazandaran province and a mountainous region were selected to conduct TCS. Details of the PERSIAN cohort and TCS have been presented in previous studies.^20-22^

 In the first phase of TCS, 10,255 participants aged 35–70 years were recruited from urban and mountainous areas in Sari, Mazandaran, Iran (7,012 urban and 3,243 mountainous populations). A subset of data (4,149 individuals, all men who participated in the TCS) was utilized in the present study. In the Tabari Cohort dataset, the number of female participants reporting opium consumption was very low, which limited the feasibility of performing reliable subgroup analyses for women. To ensure sufficient statistical power and a more robust interpretation of the results, our analyses were limited to male participants, among whom opium use was considerably more prevalent. This decision also helped reduce heterogeneity related to gender-based behavioral and cultural differences in substance use.

###  Data collection

 The survey consisted of a questionnaire, blood collection, and BP measurement.

###  Instruments

 A standardized questionnaire developed by the PERSIAN cohort team was used for data collection. The questionnaire comprises 482 items divided into general, medical, and nutritional sections. A trained interviewer administered each part of the questionnaire, which included questions on demographic information, socioeconomic status, occupational history, fuel type, lifestyle, history of chronic diseases, and opium consumption history. Other questions were related to family history, oral health, sleep status, physical activity, smoking and drinking habits, food frequency, food supplements, drinking habits, dietary habits, and exposure to pesticides. Participants were asked about their history of opium use, and their mode (oral, inhalation, or other) and duration (week, month, or year) were recorded if they reported its use.

###  Blood collection

 Fasting blood sugar (FBS) levels were measured after a 12-hour fasting period using a fully automated chemistry analyzer, the BIOTECNICA BT-1500.

###  Blood pressure measurement

 Participants’ BP was measured according to the PERSIAN cohort protocol by two trained nurses using Riester barometers. BP was assessed twice over a 10-minute interval.

###  Definitions and statistical analysis

 HTN was defined as systolic BP ≥ 140 mm Hg, diastolic BP ≥ 90 mm Hg, a history of HTN diagnosis, or taking antihypertensive medications among participants free from cardiovascular diseases. DM was defined as FBS ≥ 126 mg/dL, a history of diagnosis, or taking glucose-lowering medications among all participants.

 Statistical analyses were conducted using SPSS, version 22. Continuous variables were reported as means and standard deviations (SD), and categorical variables were presented as proportions. Chi-squared and independent t-tests were utilized to compare categorical and continuous variables, respectively, between opium users and non-users. Additionally, a multivariate logistic regression analysis was performed to adjust for potential confounding factors.

 In the first phase of the PERSIAN Cohort Study, data were collected using an intelligent and standardized electronic system. All data entries were initially reviewed by local supervisors and subsequently verified by the central scientific committee, resulting in a very low rate of missing data.

 Nevertheless, the percentage and pattern of missing data were thoroughly assessed before analysis. When the proportion of missing values for a given variable was less than 5%, a complete case analysis was applied to handle the missing data.

## Results

 Data from 4,149 male participants were included in the current study. Among them, 584 (14.1%) were opium users, while 3,265 (85.9%) had no history of opium consumption. Table 1 presents the demographic and clinical variables of opium consumers and non-users. Significant differences were observed in the education level, socioeconomic status, place of residence, and body mass index (BMI) between the two groups (*P*< 0.001).

**Table 1:** Demographic and Clinical Variables in Opium Consumers and Non-Users

	**Total (4149)**		**With opium** **consumption, n=584**		**Without opium** **consumption, n=3265**		***P*** ** value**
**Categorical variables**	**Number**	**Percent**	**Number**	**Percent**	**Number**	**Percent**	
Age (year)							
35-39	586	14.1	80	13.7	506	14.2	0.240
40-49	1282	30.9	192	32.8	1090	30.6	
50-59	1360	32.8	172	29.5	1188	33.3	
60-70	921	22.2	140	24.0	781	21.9	
Marital status							
Married	4086	98.5	573	98.1	3213	98.5	0.436
Single/divorced/widow	63	1.5	11	1.9	52	1.5	
Educational level							
Illiterate	331	7.5	48	8.2	263	7.4	0.001
1-5	674	16.2	113	19.3	561	15.7	
6-8	503	12.1	103	17.6	400	11.2	
9-12	1328	32.0	212	36.3	1116	31.3	
Academic	1333	32.1	108	18.5	1225	34.4	
Socio-economic level							
1	653	15.7	132	22.6	521	14.6	0.001
2	797	19.2	136	23.3	661	18.5	
3	815	19.6	103	17.6	712	20.0	
4	915	22.1	108	18.5	807	22.6	
5	969	32.4	105	18.0	864	24.2	
Place of residence							
Urban	2946	71.0	341	58.4	2605	73.1	0.001
Rural	1203	29.0	243	41.6	960	26.9	
Body mass index (kg/m^2^)							
< 25	1400	33.7	262	44.9	1138	31.9	0.001
25-29.9	1896	45.7	232	39.7	1664	46.7	
≥ 30	853	20.6	90	15.4	763	21.4	
Diabetes							
No	3503	84.4	490	84.0	3013	84.5	0.700
Yes	646	15.6	94	16.0	552	15.5	
Hypertension							
No	3391	81.7	482	82.5	2909	81.6	0.588
Yes	758	18.3	102	17.5	656	18.4	
Systolic hypertension							
No	3932	94.7	556	95.2	3376	94.7	0.610
Yes	217	5.3	28	4.8	189	5.3	
Diastolic hypertension							
No	3980	95.9	563	96.4	3417	95.8	0.529
Yes	169	4.1	21	3.6	148	4.2	
Continuous variables	**Mean**	**SD**	**Mean**	**SD**	**Mean**	**SD**	***P*** **value**
FBS (mg/dL)	110.34	33.89	107.73	32.19	110.77	34.14	0.048
Systolic BP (mm Hg)	115.70	13.60	114.95	13.51	115.82	13.61	0.152
Diastolic BP (mm Hg)	73.87	7.77	73.59	7.86	73.91	7.73	0.363

*Note*. SD: Standard deviation; FBS: Fasting blood sugar; BP: Blood pressure.

 The prevalence of DM among opium consumers and non-users was 16% and 15.5%, respectively (*P*= 0.705). Additionally, the prevalence of HTN among opium consumers and non-users was 17.5% and 18.4%, respectively (*P*= 0.588). FBS levels were calculated for both groups, with values of 110.77 (34.14) for opium users and 107.73 (21.19) for non-users (*P*= 0.048). Systolic BP (SBP) measured 115.70 (13.60) for opium consumers and 114.95 (13.51) for non-users (*P*= 0.152), while diastolic BP (DBP) recorded 73.59 (7.86) and 73.91 (7.73), respectively (*P*= 0.363), the related data are provided in Table 1.

 Logistic regression analysis revealed a non-significant 5% increased risk of developing DM among opium consumers (odds ratio [OR]: 1.05; 95% confidence interval [CI]: 0.82 to 1.33; *P*= 0.705). Multivariable analysis indicated a clinically notable increase in the odds of developing DM (OR: 1.22; 95% CI: 0.95 to 1.57; *P*= 0.116). In terms of HTN risk, logistic regression analysis demonstrated a non-significant reduction among opium consumers (OR: 0.94; 95% CI: 0.75 to 1.18; *P*= 0.588). However, multivariable analysis showed a slight increase in HTN risk (OR: 1.06; 95% CI: 0.82 to 1.35; *P*= 0.667), the details of which are presented in Table 2.

**Table 2:** Odd ratios of developing diabetes and hypertension in opium addicts

	**OR (95% CI)**	***P*** ** value**
Diabetes		
Crude	1.05 (0.82, 1.33)	0.705
Model 1	1.15 (0.90, 1.48)	0.253
Model 2	1.22 (0.95, 1.56)	0.121
Model 3	1.22 (0.95, 1.57)	0.116
Hypertension		
Crude	0.94 (0.75, 1.18)	0.588
Model 1	1.03 (0.80, 1.32)	0.785
Model 2	1.05 (0.82, 1.34)	0.682
Model 3	1.06 (0.82, 1.35)	0.667

*Note*. OR: Odds ratio; CI: Confidence interval; BMI: Body mass index. Model 1: Adjusted for age and BMI. Model 2: Adjusted for age, BMI, and place of residence. Model 3: Adjusted for age, BMI, place of residence, educational level, and socio-economic level.

 The analysis of variance revealed no significant differences in mean FBS, DBP, and SBP across three categories of opium consumption (oral, inhalation, and combined methods; *P*= 0.930,* P*= 0.602 and* P*= 0.565, respectively). Although no significant relationship was observed between DM and the method of opium consumption, the prevalence of HTN was significantly lower among participants who used opium via inhalation (*P*< 0.001).

 The comparisons of mean FBS, DBP, and SBP between participants whose opium consumption occurred a week, month, or year ago also represented no significant differences (*P*= 0.099, *P*= 0.150, and* P*= 0.401, respectively). Similarly, no significant associations were found when comparing the prevalence of DM and HTN across these groups (*P*= 0.155 and *P*= 0.527, respectively).

## Discussion

 Our findings demonstrated no significant differences in the prevalence of DM and HTN between opium consumers and non-users. Furthermore, the proportions of diastolic and systolic HTN were compared between the two groups, and the results showed no significant differences. Although FBS levels in opium consumers were significantly lower than those in participants without opium consumption, after adjusting for potential confounders, there were no statistically significant differences in the risk of developing DM among opium consumers. A similar pattern emerged in the multivariate analysis when assessing the risk of developing HTN.

 Azod et al^16^ conducted a study in the central region of Iran, comparing FBS, 2-HPP (2-hour postprandial blood glucose), and hemoglobin A1C levels between opium addicts and a control group. Their findings revealed that FBS was significantly lower in opium consumers. However, 2-hour postprandial blood glucose and hemoglobin A1C levels were similar in both groups, which conforms to our results. Furthermore, Dehghani et al^23^ also reported similar results. To compare the aforementioned study with the present study, the applied methodology should be taken into consideration. Dehghani and colleagues’ study had a case-control design that evaluated a limited number of participants.

 Hoseini et al^24^ demonstrated that there was no significant difference in FBS between opium users and the control group, which is in contrast to our findings. This difference in results may be attributed to the gender distribution of the studies. Our study focused on male participants, whereas the above-mentioned study evaluated a population consisting of more than half female participants. Najafipour et al^5^ investigated the prevalence of opium consumption and its association with cardiovascular disease risk factors and found that occasional opium users had a 10% increased risk of developing DM, although this increase was not statistically significant. Moreover, there was no change in the risk of DM among opium-dependent participants. Our findings also indicated an elevated risk of developing DM due to opium consumption. The slight differences between the odds ratios reported by Najafipour et al and our study may be attributed to the variables considered for adjustment in the multivariable analysis. In our study, the full model adjusted for the ORs based on age, BMI, place of residence, education, and socioeconomic level. In contrast, Najafipour et al considered age, gender, and risk factors for cardiovascular diseases, including lipid profile, physical activity, and socioeconomic status, for adjustment.

 Despite numerous studies investigating the impact of opium abuse on serum glucose levels, no consensus has been reached. To the best of our knowledge, this study represents one of the most comprehensive investigations on this topic, confirming that opium abuse has no significant effect on the development of DM.

 Some studies have examined the prevalence of HTN in opium consumers compared to non-addicts.^5,23^ Dehghani et al demonstrated that the prevalence of HTN was lower among opium-dependent participants. Likewise, our results indicated a similar pattern, although this finding was not statistically significant in our study. In a study conducted by Najafipour et al, there was a 10% reduction in the risk of developing HTN among occasional opium consumers. In contrast, opium-dependent participants showed a 10% increase in HTN risk. However, neither of these mentioned ORs was statistically significant. Our investigation illustrated a 6% risk reduction in univariate analysis and a 3–6% increased risk of HTN in multivariable logistic regression.

 Maino et al^25^ observed that the initial adjusted risk of developing HTN among opium abusers was initially 11.7 times higher. However, after considering BMI as a cofounder and including it in the multivariable analysis, the risk was reduced 9-fold. The significant difference between the adjusted odds of developing HTN in the mentioned study and our investigation could be attributed to the differences in the inclusion criteria used in the studies. Maino et al evaluated patients with a history of cardiac disease, whereas our sample resembled that of the general population. This finding suggests the potential impact of chronic disease on the risk of developing HTN in opium addicts. Ebrahimi et al^26^ found an elevated prevalence of HTN among opium consumers. Conversely, our findings revealed no significant difference in HTN prevalence between opium-dependent and non-opium user groups. Nalini et al^27^ investigated the incidence of DM and its association with opium use. Their findings are in line with ours, as their study showed lower SBP and DBP in opium users and a lower prevalence of HTN in this group.

 The influence of cultural acceptance of opium use, variations in sample size and geographic region, and differences in the definition of opium dependency across studies were considered in the current study to provide a more critical perspective. These contextual factors may partly explain why some studies have demonstrated the protective effects of opium, while others have reported harmful consequences. Moreover, our findings align with those of some studies, suggesting a neutral or non-significant association between opium use and chronic conditions, such as DM and HTN. This could be due to the dominant role of confounders, such as age, BMI, socioeconomic status, and pre-existing lifestyle factors, which we carefully adjusted for in our analysis. Studies that failed to adjust for these may have overestimated the effects of opium use.

 Our findings have important implications for public health. In regions where opium use is culturally normalized or medically misunderstood, health professionals should address misperceptions about the “therapeutic” role of opium. Clinical guidelines should emphasize the lack of protective metabolic effects and the potential risks of opium consumption. From a policy standpoint, addiction screening could be integrated into routine assessments for chronic diseases, especially in middle-aged populations. It is also recommended that future researchers hold community-based educational campaigns targeting the misconception that opium use benefits patients with HTN or diabetes.

 The present study had several limitations.First, due to the cross-sectional design of this study, we could not establish any causal relationships between opium consumption and the outcomes of DM or HTN. The findings should, therefore, be interpreted with caution and considered associations rather than cause-effect conclusions. Second, the study included only male participants, which limits the generalizability of the findings to women. Third, self-reported data on opium use may be subject to recall bias or underreporting due to social stigma. Accordingly, future longitudinal studies are needed to confirm our findings and explore potential biological mechanisms underlying the associations.

**Highlights**

- The risk of developing diabetes did not differ significantly between opium users and participants who did not use opium.
- The risk of developing hypertension (HTN) did not differ significantly between opium users and those who did not use opium.
- Fasting blood sugar level was significantly higher in opium users.

## Conclusion

 According to the findings of the present study, opium addicts were not found to be at a higher risk of developing DM and HTN. Nevertheless, given the high prevalence of opium use and its known adverse effects on other health domains, it remains essential to implement public health interventions and awareness programs targeting substance use, even if no direct association with DM and HTN was observed in our analysis.

## Acknowledgments

 This study was funded by the Iranian Ministry of Health and the Research Deputy of Mazandaran University of Medical Sciences. We gratefully acknowledge the invaluable contributions of the participants and the dedication of the PERSIAN and Tabari cohort staff

## Competing Interests

 The authors declare no conflict of interests.

## Ethical Approval

 The research protocol was approved by the Ethics Committee of Mazandaran University of Medical Sciences (IR.MAZUMS.RIB.REC.1402.022). Furthermore, written informed consent was obtained from all participants prior to data collection, in accordance with the Declaration of Helsinki.

## Funding

 Not applicable.
